# Supplementary material for: Are carbon emissions trading and green financial instruments synergistic? -Comprehensive quantitative research based on content analysis
Source: PLoS One. 2024 Mar 7;19(3):e0298601. doi: 10.1371/journal.pone.0298601 (PMC10919601; doi:10.1371/journal.pone.0298601)
Supplement: S1 Data — (ZIP) [file pone.0298601.s001.zip › Data/地级市Z_F工作报告.zip!/╜¡╦╒/╠⌐╓▌╩╨/└┤╫╘╣1⁄2╓┌║┼í╛┬φ┐╦╩2╛▌═°í┐_2019.docx]

各位代表：

现在，我代表市人民政府向大会报告工作，请予审议，并请市政协委员和其他列席人员提出意见。

一、2018年工作回顾

过去的一年，我们把深入学习贯彻习近平新时代中国特色社会主义思想、党的十九大精神和习近平总书记对江苏工作系列重要讲话指示精神作为统领一切工作的总纲，在中共泰州市委的正确领导下，全面落实党中央、国务院和省委、省政府决策部署，坚持稳中求进工作总基调，认真贯彻新发展理念，着力打好“三大攻坚战”，全力做好“六稳”工作，致力交好新时代“五张新答卷”，统筹推进稳增长、促改革、调结构、惠民生、防风险、保稳定各项工作，实现高质量发展良好开局。地区生产总值突破5000亿元，达5107.6亿元，增长6.7%；一般公共预算收入366.6亿元，增长6.6%；固定资产投资增长9.2%；社会消费品零售总额增长5.8%；城乡居民人均可支配收入分别达43452元、21219元，分别增长8.5%、8.8%；居民消费价格涨幅2%；城镇登记失业率1.78%。

经济运行稳中有进。积极应对复杂形势，始终保持发展定力，不断提升经济发展质量和效益。产业转型步伐加快。大健康产业集聚发展规划正式获批，五大主导产业产值增长11.6%，造船完工量居全国地区排名之首，被省政府表彰为制造业创新转型成效明显市。获批工信部绿色制造示范单位7家，淘汰属落后生产工艺的中（工）频炉1091台，单位地区生产总值能耗下降3.7%，主要污染物排放量削减完成省定目标。实现建筑业总产值3681.2亿元，国家特级资质企业达8家。服务业增加值增长7%，新增省级生产性服务业集聚示范区2家，泰州金融广场基本竣工并启动运营。旅游业总收入超过360亿元，全年接待游客2792万人次。企业发展总体平稳。规模以上工业产值、增加值分别增长9.8%、5.5%，实现主营业务收入6980亿元、利润455.5亿元，工业一般纳税人开票销售增长12.6%。获评省“专精特新”小巨人企业13家、国家制造业单项冠军示范企业2家，扬子江药业集团营业收入突破800亿元、列中国企业500强第242位。降低实体经济成本工作全省领先，新增各类市场主体8.8万户，被普华永道等权威机构评为全国营商环境质量指数排名提升最快的十个城市之一。加大金融服务实体经济力度，新增新三板挂牌企业3家，普惠金融领域贷款增长12.7%，市产融综合服务中心注册上线企业超过1.2万家。有效投入继续扩大。实施“236”招商计划，落实“五个一”督查推进机制，亿元以上项目新签约1468个、新开工518个、新竣工407个。21个项目入选省重点工业投资项目计划，圣达高精超薄铜箔、中来光电二期等一批重大项目竣工投产。实施智能化改造升级项目315个，创成省级示范智能车间8家，新增工业机器人490台、上云企业1165家。

重大部署有序展开。按照中央、省关于打好“三大攻坚战”的部署要求，集中力量，精准施策，在补好短板的同时“加固底板”。风险防范扎实推进。建立金融风险监测预警系统，稳妥处置“僵尸企业”、去产能企业债务风险，不良贷款率、规上工业企业资产负债率分别降至1.44%、52.5%，非法集资刑事发案数、涉案金额、涉案人员实现“三下降”。获批取消企业银行账户开户许可证核发国家级试点，在全国率先试行人民币跨境结算便利化“绿色通道”，企业债券发行工作获国务院表彰。按照“清、规、控、降、防”的要求，实行政府债务限额管理，严格控制新增债务，通过财政资金安排、有效资产注入等方式妥善化解存量隐性债务。扶贫脱贫成效彰显。聚焦黄桥老区和里下河经济薄弱地区，深入推进“阳光扶贫”工作，86.5%的建档立卡低收入人口和150个经济薄弱村实现脱贫。针对低收入人口因病致贫占比高的问题，全面实施“先诊疗后收费”政策，全年安排2000万元财政资金用于健康扶贫补充保险。突出增强经济薄弱村造血功能，市级财政投入2930万元用于村级扶贫增收项目。对口支援、扶贫协作取得重大进展，助力西藏曲水实现脱贫摘帽，完成援疆项目投入7044万元，实施咸阳扶贫协作产业项目17个。污染防治主动攻坚。抓好中央环保督察“回头看”交办问题整改，实行党政领导包案负责制，对280件交办信访件逐一过堂、对账销号，整改完成率85%。落实向环境污染宣战大会部署，成立督察百人团，九大环境专项治理取得明显成效。突出抓好长江生态修复与保护工作，拆除长江岸线违法占用项目15个。深入推进“263”专项行动，减少化工生产企业83家，完成“两断三清”171家，撤销化工集中区1家。实施大气治理工程492个，PM2.5平均浓度下降5.1%，空气质量优良天数比例提高1.3个百分点，成为全省三个“双达标”市之一。全面推行河长制，国家水生态文明城市建设试点通过验收，省考及以上断面优于Ⅲ类水质比例居全省首位。完成固废问题点位整改销号174处，城乡生活垃圾无害化处理率100%，畜禽养殖污染治理率96.1%。全域纳入淮河生态经济带发展规划，海陵、高港创成省级生态文明建设示范区。

新旧动能加快转换。统筹推进改革创新，不断扩大对外开放，进一步增强发展内生动力。重点改革落实有力。率先设立“一站式”企业开办专区，推行个体工商户简易登记，“证照分离”改革事项扩至106个，“互联网+政务服务”实现全覆盖。编制国资国企改革发展中长期规划，推进国有企业市场化转型和混合所有制改革，开展行政事业单位经营性资产和对外投资企业划转工作，率先建成国资国企监管与债务防控信息化平台，市属国有企业营业收入、利润总额分别增长21.4%、53.6%。实施“标准地”改革，开展工业项目用地综合评估，泰兴土地节约集约利用工作连续两年获国务院表彰。创新能力明显增强。落实省“科技改革30条”，开启“新时代科技新长征”，全社会研发经费支出占比2.55%，高新技术产业产值占比提高1.2个百分点。推进品质泰州建设，荣获第三届中国质量奖提名奖，获批创建标准国际化创新型城市试点。新增省级工程技术研究中心14家、企业技术中心27家，企业参与制修订国际标准3项。新型研发机构签约落户8家、投入运营5家，创成省级众创空间2家、孵化器7家。发明专利授权量增长34.2%，新增省重点研发计划项目16个。出台“科技人才新政80条”，新引进高层次人才2318名，入选省“双创”人才24名。对外开放持续深化。积极应对中美贸易摩擦影响，实现进出口总额147.3亿美元，增长13.8%。实际利用外资15亿美元，其中战略性新兴产业利用外资增长20%、占比提高8个百分点。新批境外投资项目35个，完成外经营业额9.1亿美元。医药高新区获批建设国家创新型特色园区，靖江获批设立保税物流中心（B型），黄桥工业园区升级为省级经济开发区，推动建设江阴—靖江高质量跨江融合发展实验区。海关、海事、边检为开放开发做出积极贡献。

城乡发展协调推进。开展2035版城市总体规划和城市总体设计编制，突出中心城市十大开工、十大竣工、十大在建项目建设，抓好乡村振兴战略的贯彻落实，加快城乡融合发展步伐。城市功能环境加速提升。328国道海姜段快速路正式通车，东环高架北延、永定路西段、站前路快速化改造有序推进。红旗大道东延全线贯通，鼓楼路、春兰路、祥泰路南延等主次干道建成通车，莆田路南延、运河路西延、开发路东延等“微循环”项目完工。汽车客运西站项目竣工，关帝庙地下立体停车库投入使用。智堡公园、凤凰河海棠岛建成开放，创成省生态园林城市。完成黑臭水体达标整治11条，铺设污水管网71.5公里。新辟优化公交线路11条，新增停车位5000个。建成71个便民服务“城市港湾”，农贸市场提档升级完工率84%。重大交通基础设施进一步完善。北沿江高铁完成预可研审查，正式进入工可研究阶段，常泰过江通道开工建设。京沪高速江广段扩容工程建成通车，5条高速公路前期工作加快推进。长江崩岸应急治理工程完工，马甸水利枢纽、川东港整治通过验收。泰州港吞吐量达2.45亿吨，增幅居全省首位。扬泰国际机场完成一期改扩建，飞行区等级提升至4E级。乡村振兴战略稳步实施。深化农业供给侧结构性改革，新建高标准农田30万亩，新培育家庭农场850家、为农服务综合体45家，农业适度规模经营比重86.5%，获省粮食安全责任制考核优秀等次。市农业开发区通过国家农业科技园区创建验收，高港通过国家农产品质量安全区创建验收，兴化、姜堰获批创建全国农村一二三产业融合发展先导区。入围国家新型城镇化标准化试点，特色田园乡村建设考核全省第一，碧水东罗农民丰收节列入全国系列、全省重点活动。完成农村户厕无害化改造12778座，铺设乡镇污水管网134公里，疏浚乡村河道636条，改造农村道路505公里、桥梁312座、危房1389户。农村集体“三资”监督管理创新做法全省推广，“农牧旺”、小杨村家庭农场建设等入选全省基层改革创新案例。

人民生活持续改善。坚持以人民为中心的发展思想，突出保基本、补短板、兜底线、促公平，不断满足人民美好生活需要。富民惠民力度加大。支持重点群体创业就业，发放创业担保贷款8.1亿元，新增创业9万人、就业10.4万人。推进城乡居民基本医疗保险市级统筹，生育与职工医疗保险合并实施试点经验全国推广，提前两年实现城乡低保标准一体化。市区基本建成保障性住房3068套，上争棚户区改造补助资金2.5亿元。全省首创养老服务“三张券”，“泰有福”养老服务平台建成运营，居家养老上门服务惠及14万老年人，夕阳红中心开放接待9万人次。公共服务供给优化。制定老通扬运河（古盐运河）文化带建设三年行动计划，发放文化惠民券1000万元，超额完成基层综合性文化服务中心年度建设任务，泰州科技馆参观人次超过30万。新（改扩）建幼儿园及中小学79所，普通高中扩招2248人，增加优质学位2.6万个；学前教育省优质园比例连续四年居全省首位，义务教育学校建设达标率提高8.3个百分点，三星级以上优质高中占比全省第一，新创省四星级高中3所，泰州职业技术学院通过省级示范性高职院校验收，高等教育“135”工程取得突破性进展。开展24家公立医院薪酬制度改革试点，医用耗材集中采购模式全省推广，市中心血站、急救中心建成投用，顺利通过国家卫生城市复评。成功申办第二十届省运会，泰州远大足球俱乐部冲进中乙联赛。做好双拥优抚安置工作，保障退役士兵和优抚对象合法权益。民族宗教、社会科学、广播电视、妇女儿童、残疾人、史志档案工作取得新进步，统计、气象、民防、台务、外事侨务工作取得新成绩。社会保持安定和谐。创新社会治理模式，网格化社会治理走在全省前列。加强“平安泰州”建设，推进扫黑除恶专项斗争，万人刑事案件、八类案件发案率保持全省最低，群众安全感、对政法机关和政法队伍满意率名列全省前茅。完善信访矛盾化解机制，率先出台信访人失信惩戒办法，进京访大幅下降，非访保持全省低位。深化“七五”普法，开展宪法宣传“124”行动。建立“一体四中心”司法援助体系，成为全国农民工法律援助品牌建设示范点。加强食品药品安全监管，食品安全基层机构快检室标准化建设实现全覆盖，仿制药一致性评价工作成效明显。推进省安全生产领域改革发展试点，安全生产事故数、死亡人数分别下降7.9%、3.5%。

自身建设不断加强。坚持聚焦发展，致力提高效能，有序推进党政机构改革，专题开展市级机关部门规范履职情况督查，务求政府履职有限有为有责有效。坚持依法行政。完成重大行政决策事项合法性审查33项，提请市人大常委会审议地方性法规草案2件，成立城市建设管理委员会，促进依法、科学、民主决策。实施行政执法“三项制度”，办结行政复议案件210件。落实向人大报告、向政协通报制度，完善建议提案评估交办、协商办理、督导考核机制，兴化人民饮用长江水、规范校外培训机构和治理有偿家教等建议提案得到有效落实。建立政务诚信监督体系，坚持政府部门定期新闻发布会制度，获评全省政务公开工作先进单位。突出规范高效。出台坚持勤俭节约过紧日子的意见，制定政府投资项目管理和跟踪审计办法，建成财政专项资金阳光监管平台，严格预算控制，压缩一般性支出，提高财政资金使用效益。统筹规范督查检查考核工作，加强联动督查、媒体督查，提升政府系统执行力落实力。制定实施进一步提高政府工作运行效率的意见，创新设立“骏马奖”，健全完善“三项机制”，提振干事创业精气神。改进工作作风。深入开展“解放思想再出发、对标找差新跨越”大讨论活动，出台高质量发展实施意见及系列三年行动计划，在解放思想中务实奋进。扎实开展大走访大落实新风行动，精心组织营商环境互查暗访，注重通过12345热线、市长信箱等渠道解决群众诉求，在服务基层中不忘初心。认真开展中央、省委巡视反馈意见整改工作，严格落实中央八项规定及其实施细则精神，着力整治侵害群众利益的不正之风和腐败问题，在廉洁从政中树好形象。

各位代表，一年来的成绩来之不易！这是以习近平同志为核心的党中央坚强领导的结果，是习近平新时代中国特色社会主义思想在泰州生动实践的结果，是省委、省政府和市委正确领导的结果，是市人大、市政协及社会各界关心支持的结果，是全市人民团结拼搏、奋发作为的结果。在此，我代表市人民政府，向全市广大干部群众，向全体人大代表、政协委员，向各民主党派、工商联、无党派人士、各人民团体和广大离退休老同志，向中央和省驻泰机构以及驻泰部队指战员、武警官兵和政法干警，向所有关心支持泰州发展的海内外朋友们，表示衷心的感谢！

我们也清醒地认识到，经济社会发展中还存在不少困难和问题：一是经济下行压力加大。投资增速持续放缓，加之高基数影响，增长空间更加有限；改善类商品消费呈下降态势，安心放心的消费环境仍需完善，居民消费潜力有待激发；中美贸易关系走势不明，外经外贸形势复杂。二是企业经营困难较多。融资难融资贵问题尚未得到有效解决，中小企业融资成本相对偏高；生产成本持续上升，环境治理成本有所增加，部分企业盈利空间受到压缩；市场环境波动起伏，需求结构快速调整，不少企业面临生存和发展压力。三是产业层次有待提高。“高端产业、低端环节”现象普遍存在，传统产业转型升级艰难复杂，新经济发展尚处于起步阶段；高层次研发机构和创新平台仍较缺乏，人才、资金、技术等创新要素相对不足；基地型、龙头型、引领型项目数量偏少，开发园区体制不活、效率不高。四是风险隐患不容忽视。企业债务和担保圈风险化解面临不少困难，部分领域不良贷款存在反弹压力，政府债务风险总体可控但化解压力较大；化工、不锈钢等重化工业污染点位底数不清，历史遗留问题情况复杂，处置能力相对欠缺，环境风险时有暴露；安全生产、信访维稳、社会治安等领域依然存在不少隐患。五是城市能级亟需提升。高铁、过江通道等重大交通基础设施短板明显，教育、医疗、养老等公共服务设施与群众需求仍有差距；城市经营还处于探索阶段，市场主体和社会资本在城市建设中的参与度不高；交通出行不便、环境脏乱差等问题仍较突出，提高城市管理水平需加倍努力。六是营商环境还要优化。为企服务形式、内容、机制仍需完善，部分审批事项依然存在材料多、时间长、收费高等问题，办事效率和透明度有待进一步提高；政策落实不到位、相互之间不协调，也增加了企业成长的烦恼；少数政府公务人员仍存在“庸懒散慢拖”问题，担当作为精神、干事创业劲头有所弱化。对于这些问题，我们一定认真予以重视，切实加以解决。

二、2019年工作任务

今年是新中国成立70周年，是决胜高水平全面建成小康社会的关键之年，也是全力打造江苏高质量发展中部支点城市的起始之年，做好今年政府工作任务艰巨、意义重大。

今年政府工作的总体要求是：坚持以习近平新时代中国特色社会主义思想为指导，全面贯彻党的十九大和十九届二中、三中全会精神，深入落实习近平总书记对江苏工作系列重要讲话指示精神和中央经济工作会议、省委十三届五次全会、省十三届人大二次会议、市委五届六次全会部署安排，坚持稳中求进工作总基调，坚持新发展理念，坚持推动高质量发展，坚持以供给侧结构性改革为主线，坚持深化市场化改革、扩大高水平开放，继续打好“三大攻坚战”，扎实做好“六稳”工作，保持经济持续健康发展和社会大局稳定，为高水平全面建成小康社会收官打下决定性基础，全力打造江苏高质量发展中部支点城市，奋力书写新时代“五张新答卷”，以优异成绩庆祝中华人民共和国成立70周年。

今年全市国民经济和社会发展主要预期目标为：地区生产总值增长7%左右，一般公共预算收入增长5%左右，固定资产投资增长6%，社会消费品零售总额增长7%左右，城乡居民收入与经济同步增长，居民消费价格涨幅3%左右，城镇登记失业率控制在3%以内，全社会研发经费支出占比2.6%，节能减排完成省定目标。

今年将重点抓好十个方面的工作：

1. 优化营商环境，扶持民营经济实现更大发展。认真落实习近平总书记在民营企业座谈会上的重要讲话精神，真正把民营企业和民营企业家当成自己人，与企业一道翻越市场的冰山、融资的高山、转型的火山，营造企业有活力、发展有效率的“小气候”。全面推行公平竞争审查制度，清理违反公平、开放、透明市场规则的政策文件，精简涉及民间投资管理的审批事项，让企业在合法合规的前提下理性决策、自主发展。多措并举为企业尤其是小微企业实质性减负，落实普惠性税收免除和降低社会保险费率政策，加快推进涉企行政事业性收费零收费，规范中介服务收费和经营服务性收费，力争全年再降低企业成本50亿元。推动“银政企保”合作和银税互动，完善政策性融资担保、应急转贷、风险补偿体系，鼓励风险投资、股权投资、债券发行机构发展，进一步提高制造业新增贷款和直接融资比重。做好第四次全国经济普查及数据开发利用，构建普惠化、功能性产业政策体系，防止政策执行“差别化”“一刀切”，确保惠企利企举措落到实处，更好地稳定和引导市场预期。紧扣广大企业和创业者的实际需求，谋划建设“企业服务云”平台，让为企服务又好又快、政商关系“清”上加“亲”。全面落实中央、省关于完善产权保护制度依法保护产权的实施意见，妥善处理民营企业产权纠纷和历史问题，让广大企业家吃下定心丸、安心谋发展。实施“十佳百强千优”企业行动计划，激发和弘扬企业家精神，培育一支有立场、有情怀、有坚守、有匠心的企业家队伍。

2. 攻坚重大项目，为构建现代产业体系夯实根基。始终把重大项目建设置于“头版头条”，组织实施项目大提升三年行动计划，以有效投入助推产业转型、提高发展质量。围绕构建“1+5+1”现代产业体系，加强对优势主导产业的细分研究，强化市域统筹，注重错位发展，加快形成“一带两块”产业发展格局。探索建立“亩产论英雄”的综合评价体系，明晰新上项目设备投资额、投资强度、单体规模、投入产出比等标准，力争单位建设用地税收收入达4.7亿元。全力招引处于产业前沿、拥有核心技术、具备领先优势的行业龙头企业，推动本土企业与科技类军工类央企、世界500强企业加强战略合作，力争10亿元以上项目新签约80个、新开工40个、新竣工30个。开展智能化改造年度行动计划，完善重大技改项目联系服务机制，提高数控装备、加工中心和工业机器人应用水平，组织实施重点智能化改造升级项目300个以上，新增市级以上示范智能车间30个。大力发展循环经济，严把环保、能耗、安全等项目准入门槛，新上一批节能环保、循环再生、清洁能源产业项目，促进开发园区生态化改造、产业企业绿色化转型。实施现代服务业高质量发展三年行动计划，扩大旅游、文化、体育、健康、养老等幸福产业项目投资，推进高端商贸、现代金融、现代物流、信息服务等功能型项目建设，力争服务业增加值占比提高0.5个百分点。

3. 强化科技支撑，营造富有特色优势的创新生态。必须把科技创新摆在发展全局的核心位置，加速创新链与产业链的对接融合，提升产业技术密度和科技含量，为新动能成长注入强劲动力。针对大健康产业、五大主导产业发展的技术短板和创新需求，支持龙头企业、行业协会、高校院所联合组建产业技术创新联盟，加强关键共性技术、前沿引领技术、现代工程技术攻关，力争万人发明专利拥有量达14件。构建普惠性政策、财政后补助、政府采购相结合的支持模式，改革科研成果申报和资助办法，力争企业研发经费投入占主营业务收入比重1.5%。加强创新型领军企业、“专精特新”小巨人企业培育，鼓励企业设立由高层次人才领衔的新型研发机构，全年净增国家高新技术企业70家。强化创新载体平台建设，拓展“泰科易”线上线下服务功能，打造一批高水平、专业化孵化器，力争10家以上重大研发载体投入运营。深化品质泰州建设，加快标准国际化创新型城市创建，开展高端品质认证试点，引导企业强化标准思维、质量观念、品牌意识，推动泰州制造高端化、品牌化。全面落实“科技人才新政80条”，着力招引“高精尖缺”人才和高水平创新团队，新引进高层次人才2400名。

4. 深化重点改革，聚力解决发展中的实际问题。切实把深化改革作为“突围中游”的关键一招，以再出发的勇气破除体制障碍和工作积弊，进一步激发市场活力和社会创造力。深化“放管服”改革，构建一体化网上政务服务体系，让企业和群众办事线上“一网通办”、线下“只进一扇门”，在更高层次实现“不见面审批”；推动“双随机、一公开”监管全覆盖、常态化，提高包容审慎监管、综合监管、协同监管水平，实行守信联合激励、失信联合惩戒，为守法诚信企业创造一个低成本的外部环境。强化金融风险防范化解与金融服务实体经济的统筹协调，推动金融政策、机构、平台、产品集成创新，加强外资金融机构、大型商业银行、金融服务组织招引培育，抓好地方法人金融机构组建、企业上市挂牌等工作，确保金改试点取得新的突破。加快国有企业市场化转型发展步伐，整合优质资产资源，理顺管理体制机制，提高资产质量和竞争能力；推动国有企业做大主业规模、重塑运营模式、强化资本运作，全面参与公共服务和基础设施项目建设，不断提升经济社会效益；完善国有企业法人治理结构，进一步健全监管制度、改进监管方式。坚持勤俭节约过紧日子，实施预算绩效管理改革，落实政府隐性债务化解方案，规范产业投资基金、政府和社会资本合作项目运营管理，当好人民的“理财管家”。

5. 扩大对外开放，不断提升经贸交流合作层次。主动把开放的大门越开越大，按照省委以“一带一路”交汇点建设总揽新时代江苏对外开放的部署要求，实施开放型经济高质量发展三年行动计划，加快形成全方位对外开放新格局。抢抓长三角一体化发展上升为国家战略的重大机遇，积极策应上海“五个中心建设”，主动“攀亲”，精准对接，寻求合作，更好地服务大上海、融入长三角。精心组织国际医药博览会、泰台交流合作周等投资促进活动，在先进制造业、现代服务业利用外资上扩量提质，进一步提高战略性新兴产业利用外资比重，力争实际利用外资16亿美元。加强对出口重点行业、地区、企业的引导支持，发挥出口信用保险政策效用，推动有实力有条件的企业布局建设“海外仓”和转口基地，开拓东盟、南美、非洲等新兴市场，力争实现出口80亿美元。扩大关键设备、先进技术和资源性产品进口规模，促进日用消费、医药器械、康养护理等产品进口，力争实现进口50亿美元。鼓励企业通过跨国并购、工程承包、技术合作等方式拓展境外业务，指导“走出去”企业强化合规管理，力争完成外经营业额9亿美元。全力塑造“健康名城”新地标，加快推进长江经济带大健康产业集聚发展试点，继续举全市之力建设中国医药城，着力培育标志性企业、打造标志性平台、集聚标志性人才，构筑全国大健康产业高地；推动医药高新区“二次创业”，把加快医药产业化提到最重要的位置，加强国家一类新药、重大疾病治疗新药、高端医疗器械的技术研发和产品开发，发展基因测序、分子诊疗等特色医疗服务，努力成为全市高质量发展的主阵地、主引擎。推动开发园区围绕主导产业调整规划布局、突出生产功能、优化创新生态，在园区建设、运营、招商、管理模式上探索创新，促进省级以上开发区转型升级、争先进位。

6. 完善城市功能，通过科学理性运作加快做美精致城市。致力把提高中心城市首位度作为主攻方向，实施城市功能品质特色提升三年行动计划，推行政府引导、企业主体、市场化运作的城建模式，建设和谐宜居、富有魅力、特色鲜明的现代化城市。运用“多规合一”理念抓好城市国土空间规划编制，同步推进城市总体设计编制和片区规划、专项规划研究，实现城市规划分层次、渐进式超前引导覆盖。围绕打造“一主一新”、形成“双核一特”，实施“老城复兴”“老城繁荣”工程，开展高铁枢纽片区前期工作，完善市区管理体制、城建体制、财税体制，加快市区融合发展步伐。继续实施东环高架北延、站前路快速化改造，确保永定路西段快速化改造建成通车，启动姜堰南绕城快速化改造，开展中心城区与泰兴之间的快速路工程研究；加快青年路北延、春晖路北延、京泰路南延等道路建设。深化省公交优先示范城市创建，调整优化运营时间和线路，新辟专用通道40公里，万人公交车拥有量达15标台。通过“城市双修”弥补城市欠账、促进城市更新，推进雨污分流改造和污水管网全覆盖工程，基本完成建成区黑臭水体整治计划；启动国家生态园林城市创建，抓好“1213”绿满泰州行动，提高城市绿化体系质量；加强老旧小区微整治，新建改造农贸市场9家，加快生活垃圾焚烧发电二期扩建、医疗固废处置、垃圾分类收集后端处理等项目建设。全力以赴推进北沿江高铁和盐泰锡常宜铁路前期工作，力争北沿江高铁开工建设。加快常泰过江通道建设，做好江阴第二、第三及张靖过江通道前期工作。力争阜兴泰高速二期、沪陕高速广陵至平潮段扩容工程开工建设，加快推进宁盐高速、兴东高速、盐靖高速扩容工程前期工作。积极融入全省港口一体化发展格局，加强江海联运中心港建设和沿江岸线资源整合，构建连接三大港区、主要园区的高品质集疏运体系。稳步开展智慧城市建设，打造城市治理综合运行平台，提高城市管理科学化精细化智能化水平，用绣花的功夫做精做细城市治理与服务。

7. 推动乡村振兴，打造群众认可的美丽宜居家园。坚持把实施乡村振兴战略作为新时代“三农”工作总抓手，加快推进农业农村现代化，促进农业全面升级、农村全面进步、农民全面发展。深化国家农业可持续发展试验示范区建设，继续开展粮食绿色高质高效创建活动，大力发展农牧结合循环型农业，力争种植业绿色优质农产品、畜禽生态健康养殖比重分别达70%、55%。创建国家农产品质量安全市，推进标准化生产基地、加工基地建设，完善农产品质量认证和安全监管追溯系统，打造一批区域公用品牌和特色农产品品牌。严格保护耕地和永久基本农田，扎实开展“大棚房”问题专项清理整治，全面落实地方政府粮食安全责任制，新增高标准农田25万亩。加快主要作物生产全程机械化，实施智慧农业工程和“互联网+”现代农业行动。突出抓好家庭农场和农民合作社两类经营主体发展，鼓励通过多种形式开展适度规模经营，新增市级示范家庭农场50家。继续扩大集体经营性资产“股权到人、固化到户”试点范围，完善农村承包地“三权分置”制度，建立涉农资金统筹使用平台，深化农业保险市场化改革。开展农村“四清四化四改”，整治县乡村河道1095条，完成农村户厕无害化改造2.9万座，确保40%的行政村建有生活污水处理设施、所有村达到“整洁村”标准，建成美丽宜居乡村200个以上、特色田园乡村17个。深化省级“四好农村路”示范县、示范市创建，力争镇村公交开通率100%。深入实施新风行动，建好用好新时代文明实践中心，加强农耕文化、优秀民间文化传承保护，建设邻里守望、重礼诚信的文明乡村。

8. 坚守生态底线，坚决整治环境保护突出问题。自觉把“生态优先、绿色发展”转化为实际行动，让绿色生态成为高质量发展的根本依托。认真落实中央环保督察整改要求，严格执行领导包案和整改销号制度，依法严厉打击环境违法行为。持续加大大气、水、土壤污染防治工作力度，全力攻坚区域水环境、畜禽养殖污染、扬尘污染等突出问题，力争空气质量优良率、省考及以上断面优于Ⅲ类水质比例分别达70%、80%以上。把修复长江生态作为压倒性任务，开展长江干流泰州段沿江缓冲带生态环境普查，抓紧完成“三线一单”编制，深入实施化工企业“四个一批”专项整治，加快推进长江生态环境整治修复20个重点项目建设，共同守护好我们的“母亲河”。加强对大宗工业固体废物、危险废物、主要农业废物、生活垃圾、建筑垃圾的规范化处置，争取成为全国“无废城市”试点。开工建设淮河流域重点平原洼地治理工程，营建江河湖渠生态防护林和绿色生态廊道，开展里下河国家湿地公园建设试点，实行严格的饮用水源地保护制度，支持兴化加快饮用长江水工程建设。放大大健康产业优势与生态资源优势，开发乡村休闲、湿地度假、健康养生等生态旅游产品，规划建设特色健康小镇、医养融合社区，打通绿水青山向金山银山转化通道。深化国家生态文明建设示范区创建，加快设立绿色发展指标体系，健全区域生态补偿机制，严格落实环境保护实绩公示、终身追责和自然资源离任审计制度。我们一定要像保护眼睛一样保护生态环境，决不走“先发展后治理”的路，决不做“吃祖宗饭、断子孙路”的事！

9. 补齐民生短板，让老百姓过上更好的生活。坚决把以人民为中心的发展思想落到工作中去，多办利民实事、多解民生难事，不断增强人民群众的获得感、幸福感、安全感。继续落实好创业富民30条，高质量建设一批“双创”示范基地，用创业春风吹动发展春潮，力争新增创业5万人。加强新时期产业工人队伍建设，完善终身职业技能培训机制，推动产教融合发展和“引企入教”改革，力争新增城镇就业6.5万人、农村转移劳动力1万人。谋划一批产业、教育、金融、基础设施等专项扶贫项目，实行城乡低保标准动态调整，落实低收入农户政策性兜底保障，健全精准脱贫退出机制，基本完成建档立卡低收入人口和经济薄弱村脱贫任务。进一步扩大社会保障覆盖面，开展总额控制下的复合式医疗保险付费方式改革，实施长期护理保险制度，强化社会救助和特殊人群关爱服务，让温暖的阳光照耀到每个人。巩固提升文明城市创建成果，抓好“泰有礼”全民主题实践活动；加强老通扬运河（古盐运河）文化带建设，深入挖掘红色文化资源，打造梅兰芳、泰州学派、里下河文学流派等文化品牌；加强城市社区和农村文化设施建设，完善城乡公共文化服务圈；精心组织人民海军诞生70周年“水兵母亲城”系列庆祝活动，高水平办好水城水乡国际旅游节、梅兰芳艺术节。深化教育体制机制改革，新（改扩）建幼儿园及义务教育学校38所，基本消除义务教育阶段超大班额现象，启动省高品质示范高中建设，提高在泰高校专业设置与产业发展的契合度，为孩子们创造更多享有优质教育的机会。完善现代医疗卫生服务体系，提升卫生信息化建设水平，推进紧密型医联体建设和家庭医生签约服务，深化国家中医药综合改革试验区建设，加快实施市妇幼保健院、第五人民医院等项目，力争新创省示范乡镇卫生院4个、卫生室36个。认真做好第二十届省运会筹办工作，有序推进市体育公园等场馆建设。提高城乡社区居家养老服务质量，探索养老服务社会化运营模式，力争护理型养老床位占比超过50%、接受上门服务的居家老人数占比12%。强化房子是用来住的、不是用来炒的定位，完善住房市场体系和保障体系，促进房地产市场平稳健康发展。开展新一轮全国双拥模范城创建，巩固军政军民团结局面。深化城乡和谐社区建设，大力推行“互助户治”“乡贤评议”等基层治理模式，着力构建共建共治共享格局。加强社会治理大数据“云+端”建设，织密立体化信息化社会治安防控体系，提高网络安全和信息化治理能力。深入开展扫黑除恶专项斗争，严厉打击涉黑涉恶违法犯罪活动。推进重点行业领域平安建设，深化“七五”普法和宪法学习宣传，推行“12348”信访矛盾化解模式，最大限度增加社会和谐因素。继续开展食品安全示范城市创建，积极培育新的消费热点，更好满足居民消费升级需求，让老百姓吃得放心、穿得称心、用得舒心。落实安全生产企业主体责任和部门监管责任，深入排查整治各类安全隐患，创新安全监管执法，完善应急救援体系，坚决防范杜绝重特大事故，提高防灾减灾救灾能力，维护人民群众生命财产安全。

10. 坚持勤政廉政，建设人民满意的服务型政府。持之以恒把“更加务实、更有担当、更高效率”打造成政府工作标记，扎实开展“不忘初心、牢记使命”主题教育，进一步提升为民务实清廉形象。做好政府工作必须旗帜鲜明讲政治，要树牢“四个意识”，坚定“四个自信”，忠诚践行“两个维护”，自觉在思想上政治上行动上同以习近平同志为核心的党中央保持高度一致，坚决把党对一切工作的领导贯彻到政府工作各领域各方面，确保中央大政方针和省市委决策部署不折不扣落地见效。依法行政是政府工作的“第一军规”，要结合政府机构改革和职能配置优化，明权责、定规则、抓履职，主动接受人大法律监督、工作监督和政协民主监督，深化审计监督、社会监督、舆论监督，提高政务公开标准化、信息化水平，让“讲程序、重规范、严约束”成为工作习惯。为人民服务是政府应尽的首要义务，要继续在发展经济的基础上，不断保障和改善民生，既尽力而为，又量力而行，着力保基本、增供给、强机制，把群众最关切最烦心的事一件件办妥办好。推动发展是政府肩负的重要职责，要深入谋划全局性方略、抓手性工作、创新性举措，多研究有效对策，多落实管用政策，切实把改革发展稳定各项工作做实做好，用铁的肩膀担负起时代赋予的责任。遵循规律是政府干事的重要法宝，要善于运用战略思维、历史思维、辩证思维、创新思维、法治思维、底线思维，摸准问题根源，找到最佳办法，学会综合平衡，在应对风险挑战上下好先手棋、出好关键招，增强工作的预见性、主动性、创造性。清正廉洁是政府公务人员的本分，要切实履行全面从严治党责任，认真贯彻中央八项规定实施细则精神和省市委具体办法，严格遵守廉洁从政纪律规定，扎实开展形式主义、官僚主义集中整治，严肃查处审批监管、资源开发、大宗采购、工程招投标等方面的腐败问题，干干净净当好人民公仆。

各位代表，幸福生活都是奋斗出来的，美好未来都是打拼出来的。让我们更加紧密地团结在以习近平同志为核心的党中央周围，坚持以习近平新时代中国特色社会主义思想为指导，在省委、省政府和市委的正确领导下，不畏艰难积极进取，满怀信心埋头苦干，以奋斗姿态冲刺高水平全面建成小康社会，全力打造江苏高质量发展中部支点城市，奋力书写新时代“五张新答卷”，加快建设“强富美高”新泰州，以优异成绩庆祝中华人民共和国成立70周年！
